# Financial Performance of Medical Corporations in Japan From 2016 to 2022: A Nationwide Longitudinal Analysis

**DOI:** 10.2188/jea.JE20250303

**Published:** 2026-06-05

**Authors:** Satoshi Funada, Shusuke Hiragi, Michiko Ashizawa, Rei Goto

**Affiliations:** 1 Health Technology Assessment Unit, Department of Preventive Medicine and Public Health, Keio University School of Medicine, Tokyo, Japan; 2 Institute of Business and Accounting, Kwansei Gakuin University, Hyogo, Japan; 3 Graduate School of Business Administration, Keio University, Kanagawa, Japan; 4 Graduate School of Health Management, Keio University, Kanagawa, Japan

**Keywords:** medical corporations, financial performance, profit margin, health system sustainability, COVID-19

## Abstract

**Background:**

Medical corporations (Iryohoujin) are central to Japan’s healthcare delivery, yet their financial conditions have not been comprehensively assessed nationwide. This study aimed to describe financial trends and structural characteristics of hospital-operating corporations from fiscal year (FY)2016 to FY2022 and examine fluctuations, including during the FY2020–FY2021 coronavirus disease 2019 (COVID-19) pandemic.

**Methods:**

A longitudinal analysis was conducted using financial data covering 95% of all hospital-operating medical corporations. Financial indicators, including total assets, medical revenue, and profit margin, were tracked from FY2016 to FY2022. Subgroup analyses were based on organizational size and integration. A linear mixed-effects model examined factors associated with profitability.

**Results:**

The number of hospital-operating medical corporations declined from 4,631 in FY2016 to 4,469 in FY2022. By FY2022, total assets and medical revenue reached 15.9 trillion Japanese yen (JPY) and 12.5 trillion JPY, respectively. Median medical profit margin declined from 1.8% (interquartile range [IQR], −1.2 to 5.3%) in FY2016 to 0.6% (IQR, −3.4 to 4.3%) in FY2021, then recovered to 1.6% (IQR, −2.5 to 5.5%) in FY2022. Smaller corporations, with fewer hospitals and beds, were more financially unstable, especially during the COVID-19 pandemic. A linear mixed-effects model showed that medical profit margin was negatively associated with hospital number and positively associated with long-term care and psychiatric beds.

**Conclusion:**

This study highlights the structural scale and financial dynamics of Japanese medical corporations, revealing a large but unevenly resilient sector in which not only hospital size but also bed composition significantly influences profitability. These findings may inform policy discussions on healthcare system sustainability and financial support.

## INTRODUCTION

In Japan, medical corporations (*Iryohoujin*) are the backbone of the healthcare delivery system, owning approximately 70% of all hospitals and managing over 55% of total hospital beds.^1^ Although privately owned, these facilities operate under strict non-profit regulations defined by the Medical Care Act.^2^ Recent analyses have shown a gradual decline in the number of medical corporations, as well as in the hospitals and beds they manage.^3^ Meanwhile, organizational restructuring through horizontal and vertical integration has slowly progressed, suggesting a gradual shift in the structural landscape of medical corporations.

Understanding the financial structure of medical corporations is critical for developing sustainable healthcare policies. In countries such as the United States, institutions like the American Hospital Association (AHA) have long provided comprehensive monitoring and reporting on hospital financial performance, offering data-driven foundations for policymaking.^4^^,^^5^ In Japan, however, most financial surveys, including those by the Japan Hospital Association (JHA), rely on voluntary responses, limiting their representativeness. A recent emergency survey revealed that 61.2% of hospitals were operating in deficit in 2024, up from 50.8% the previous year.^6^ This trend may reflect rising costs for labor, medical supplies, and utilities, as well as systemic constraints under Japan’s reimbursement system, where medical fees are set according to a nationally uniform fee schedule.^7^ This framework likely limits hospitals’ flexibility to respond to cost increases. These factors might have contributed to the observed rise in deficits; however, the interpretability of the findings remains constrained due to the reliance on self-reported data. Notably, the survey included responses from only 1,816 of the 5,901 hospitals affiliated with the JHA (30.8%), which would further limit its representativeness. While national and public hospitals disclose financial statements, medical corporations, despite accounting for the majority of hospital providers in Japan, remain understudied due to the absence of systematically collected financial data. Their financial status, market presence, and the factors influencing their performance are still poorly understood, particularly in the context of the coronavirus disease 2019 (COVID-19) pandemic.

This study aimed to address these knowledge gaps by using a comprehensive dataset of financial and structural information from medical corporations in Japan. Specifically, the objectives were to: (1) describe the overall market size and financial trends; (2) evaluate key financial indicators derived from balance sheets and income statements; (3) examine changes in financial conditions, including those observed during the COVID-19 pandemic; and (4) explore organizational characteristics associated with financial resilience.

## METHODS

### Study design

This was a longitudinal observational study based on financial and structural data from medical corporations in Japan from fiscal year (FY)2016 through FY2022. Given that this study solely utilized medical corporation data, it was exempt from institutional review board approval and participant consent requirements, in accordance with the Ethical Guidelines for Medical and Health Research Involving Human Subjects belonging to the Ministry of Health, Labour and Welfare (MHLW) in Japan.^8^ The study adhered to the Strengthening the Reporting of Observational Studies in Epidemiology reporting guidelines.^9^

### Setting

The study was conducted in Japan and examined the financial and organizational characteristics of hospital-operating medical corporations from FY2016 to FY2022.

### Data source

The dataset used in this study is based on legally mandated financial documents required under the Medical Care Act in Japan.^2^^,^^10^ Medical corporations are obligated to submit annual business reports, balance sheets, income statements, inventories of assets, and other documents specified by the MHLW to their respective prefectural governments. Submission is legally required, and non-compliance may lead to administrative penalties, such as fines. The dataset covers approximately 95% of all medical corporations in Japan and includes information on institutional characteristics, facility types, number of beds, and financial statements (balance sheets and income statements). In Japan, the FY runs from April 1 to March 31 of the following year, and these reports are aggregated accordingly. In this study, we defined FY2020 and FY2021 as the COVID-19 pandemic period.

### Study population

The target population comprised medical corporations operating hospitals in Japan. Under the Medical Care Act, medical corporations are non-profit entities that manage healthcare facilities, including hospitals, clinics, long-term care facilities, and other facilities.^2^ Other facilities refer to service providers related to health and social welfare, including community-based residential services, short-term stay facilities, day care centers, and in-home care providers. Corporations with extreme outliers in financial indicators were excluded. Further details on facility classification are described elsewhere.^3^

### Variables and definitions

#### Medical corporation characteristics

Organizational characteristics included the number of facilities by type (hospitals, clinics, long-term care facilities, and other facilities) and the number of hospital beds, categorized as general, long-term care, or psychiatric. Horizontal integration was defined as the ownership of two or more hospitals under a single medical corporation, with each hospital maintaining its own operational structure (eFigure 1). Vertical integration was defined as the ownership of a hospital along with other types of healthcare facilities, such as clinics or long-term care facilities, within the same medical corporation (eFigure 1). Based on these definitions, integration was categorized into four types: (1) horizontal integration only, (2) vertical integration only, (3) both horizontal and vertical integration, and (4) neither horizontal nor vertical integration.

#### Financial indicators

Financial indicators were extracted from balance sheets and income statements, including total assets, total liabilities, net assets, medical revenue, medical profit, ordinary revenue, ordinary profit, and net profit. Based on these financial variables, the following indicators were calculated:

• Profitability: medical profit margin, ordinary profit margin, net profit margin, return on total capital from medical business, return on assets (ROA), and return on equity (ROE)
• Liquidity: current ratio
• Stability: debt ratio, equity ratio, debt-to-equity ratio, fixed assets to long-term liabilities ratio
• Efficiency: total asset turnover

Definitions and calculation formulas for all financial indicators are provided in Table 1.

**Table 1.** Definitions and formulas of financial indicators for medical corporations in Japan

Indicator	Category	Formula
Total assets	Balance sheet	As reported in the balance sheet
Total liabilities	Balance sheet	As reported in the balance sheet
Total net assets	Balance sheet	As reported in the balance sheet
Excess liability over assets	Balance sheet	As reported in the balance sheet
Medical revenue	Income statement	As reported in the income statement
Medical profit	Income statement	As reported in the income statement
Medical profit, positive	Income statement	As reported in the income statement
Ordinary revenue	Income statement	As reported in the income statement
Ordinary profit	Income statement	As reported in the income statement
Ordinary profit, positive	Income statement	As reported in the income statement
Net profit	Income statement	As reported in the income statement
Net profit, positive	Income statement	As reported in the income statement
Medical profit margin	Profitability	Medical profit / Medical revenue × 100
Ordinary profit margin	Profitability	Ordinary profit / Medical revenue × 100
Net profit margin	Profitability	Net profit / Medical revenue × 100
Return on total capital from medical business	Profitability	Medical profit / Total capital × 100
Return on total assets (ROA)	Profitability	Net profit / Total assets × 100
Return on equity (ROE)	Profitability	Net profit / Equity × 100
Current ratio	Liquidity	Current assets / Current liabilities × 100
Debt ratio	Stability	Total liabilities / Total assets × 100
Equity ratio	Stability	Equity / Total assets × 100
Debt-to-equity ratio	Stability	Total liabilities / Equity × 100
Fixed asset to long-term liabilities ratio	Stability	Fixed assets / (Fixed liabilities + Net assets) × 100
Total asset turnover	Efficiency	Medical revenue / Total assets

### Statistical analysis

Financial indicators were described annually from FY2016 through FY2022 using counts and percentages, sums, medians, and interquartile ranges (IQRs). Subgroup analyses were conducted to compare medical profit margin by the number of hospitals, number of beds, and the presence of horizontal or vertical integration. To examine organizational characteristics associated with medical profit margin, we constructed a linear mixed-effects model with medical profit margin as the dependent variable. Fixed effects included fiscal year, number of facilities by type, number of beds by type, and integration status. Random intercepts were included for corporate identifiers to account for unobserved heterogeneity. The model specification can be expressed as:

$$\begin{array}{rl} & {\text{MedicalProfitMargin}}_{it}\\ & \;=\beta_{0}+\beta_{1}\;{\text{Year}}_{t}+\beta_{2}\;{\text{FacilityType}}_{it}+\beta_{3}\;{\text{BedType}}_{it}\\ & \;+\beta_{4}\;{\text{Integration}}_{it}+u_{i}+\varepsilon_{it} \end{array}$$

where MedicalProfitMargin*_it_* is the medical profit margin of corporation *i* in FY *t*; Year*_t_* represents FY indicators (FY2016–FY2022); FacilityType*_it_* includes the number of hospitals, clinics, long-term care facilities, and other facility types operated by each corporation; BedType*_it_* includes the number of general, long-term care, and psychiatric beds; Integration*_it_* denotes categorical indicators for integration status. The term *u_i_* ∼ N(0, *σ*^2^*_u_*) is a random intercept capturing unobserved heterogeneity across corporations; and *ε_ii_* ∼ N(0, *σ*^2^) is the residual error term. The following four models were developed: model 1 included year and number of hospitals; model 2 included year and number of total beds; model 3 included year and bed type of proportions; model 4 included year, numbers of general, long-term care, and psychiatric beds; model 5 included year and integration types; and model 6 included all variables. Model fit was assessed using the Akaike Information Criterion (AIC), Bayesian Information Criterion (BIC), and marginal and conditional R^2^ values. To assess multicollinearity among fixed effects, generalized variance inflation factors (GVIFs) were calculated prior to model estimation. All *GVIF*^1/(2×^*^Df^*^)^ values were below 3.0, indicating acceptable levels of multicollinearity. All costs were presented in Japanese yen (JPY) and converted to United States dollars (USD) using the annual average exchange rates for each fiscal year: 1 USD = 108.8 JPY in FY2016, 112.2 in FY2017, 110.4 in FY2018, 109.0 in FY2019, 106.8 in FY2020, 109.8 in FY2021, and 131.4 in FY2022.^11^ All analyses were conducted between November 2024 and April 2025 using R (version 4.4.1; R Foundation for Statistical Computing, Vienna, Austria), with the linear mixed-effects model implemented using the “*lme4*” package.^12^ Two-sided *P*-values were reported, and a threshold of *P* < 0.05 was considered statistically significant in this exploratory study.

## RESULTS

### Medical corporation trends

The study flow diagram is presented in eFigure 2. Table 2 summarizes the organizational characteristics of medical corporations from FY2016 to FY2022. During this period, the total number of corporations gradually declined from 4,631 to 4,469, accompanied by a slight decrease in the number of hospitals they operated (from 5,456 to 5,406). While most corporations continued to operate a single hospital, the proportion managing more than four hospitals increased slightly, from 1.3% to 1.6%. Likewise, the share operating 800 or more beds rose from 1.2% to 1.5%. The proportion of corporations with horizontal integration only remained stable at around 2.8% from FY2016 to FY2022, while those with vertical integration only increased from 39.6% to 42.6%. Corporations with both horizontal and vertical integration rose from 8.3% to 9.0%, whereas those with neither type of integration decreased from 49.3% to 45.6%.

**Table 2.** Annual trends in organizational characteristics of medical corporations, FY2016–FY2022

	FY2016	FY2017	FY2018	FY2019	FY2020	FY2021	FY2022
Medical corporations, *N*	4,631	4,750	4,722	4,656	4,591	4,526	4,469
Hospitals, *N*	5,456	5,626	5,628	5,596	5,531	5,466	5,406
Category by number of hospitals, *N* (%)							
1	4,118 (88.9)	4,219 (88.8)	4,180 (88.5)	4,111 (88.3)	4,055 (88.3)	3,991 (88.2)	3,941 (88.2)
2	385 (8.3)	386 (8.1)	394 (8.3)	391 (8.4)	384 (8.4)	380 (8.4)	373 (8.3)
3	70 (1.5)	82 (1.7)	80 (1.7)	85 (1.8)	80 (1.7)	84 (1.9)	82 (1.8)
4 to 9	54 (1.2)	59 (1.2)	64 (1.4)	63 (1.4)	66 (1.4)	65 (1.4)	67 (1.5)
≥10	4 (0.1)	4 (0.1)	4 (0.1)	6 (0.1)	6 (0.1)	6 (0.1)	6 (0.1)
Clinics, *N*	2,254	2,358	2,349	2,386	2,429	2,398	2,420
Long-term care facilities, *N*	1,940	2,002	2,025	2,002	2,005	1,964	1,948
Other facilities, *N*	90	88	98	184	293	390	461
Beds, *N*	820,196	843,619	847,207	836,649	823,763	812,202	799,039
Category by number of beds, *N* (%)							
<100	1,911 (41.3)	1,950 (41.1)	1,930 (40.9)	1,908 (41.0)	1,898 (41.3)	1,866 (41.2)	1,864 (41.7)
100–199	1,483 (32.0)	1,538 (32.4)	1,525 (32.3)	1,513 (32.5)	1,495 (32.6)	1,475 (32.6)	1,445 (32.3)
200–399	903 (19.5)	917 (19.3)	917 (19.4)	895 (19.2)	858 (18.7)	853 (18.8)	831 (18.6)
400–799	278 (6.0)	283 (6.0)	285 (6.0)	271 (5.8)	274 (6.0)	266 (5.9)	264 (5.9)
≥800	56 (1.2)	62 (1.3)	65 (1.4)	69 (1.5)	66 (1.4)	66 (1.5)	65 (1.5)
General beds, *N*	320,280	330,983	334,938	333,875	334,790	334,927	332,359
Long-term care beds, *N*	255,346	260,734	259,922	250,944	239,819	227,446	219,992
Psychiatric beds, *N*	244,570	251,902	252,347	251,830	249,154	249,829	246,688
Integration types, *N* (%)							
Horizontal only	129 (2.8)	135 (2.8)	142 (3.0)	130 (2.8)	124 (2.7)	124 (2.7)	125 (2.8)
Vertical only	1,835 (39.6)	1,876 (39.5)	1,871 (39.6)	1,870 (40.2)	1,909 (41.6)	1,902 (42.0)	1,905 (42.6)
Both horizontal and vertical	384 (8.3)	396 (8.3)	400 (8.5)	415 (8.9)	412 (9.0)	411 (9.1)	403 (9.0)
Neither vertical nor horizontal	2,283 (49.3)	2,343 (49.3)	2,309 (48.9)	2,241 (48.1)	2,146 (46.7)	2,089 (46.2)	2,036 (45.6)

FY, fiscal year.

eTable 1 summarizes bed composition by hospital number, bed size, and integration type. Corporations with one hospital had relatively higher proportions of psychiatric beds, whereas those with fewer than 100 beds had markedly lower proportions of psychiatric beds and higher proportions of general and long-term care beds. In contrast, the proportion of psychiatric beds increased in corporations with moderate bed capacity (200–799 beds). Corporations with both horizontal and vertical integration, as well as those operating a larger number of hospitals or beds, had the highest share of general beds and the lowest share of psychiatric beds.

### Financial indicators trends

Table 3 presents trends in financial indicators from FY2016 to FY2022. Total assets increased from 13.6 trillion JPY (125.0 billion USD) in FY2016 to 15.9 trillion JPY (121.0 billion USD) in FY2022, accompanied by a rise in median assets from 1.72 billion JPY (IQR, 0.89–3.29 billion JPY; 15.8; IQR, 8.2–30.2 million USD) to 2.00 billion JPY (IQR, 1.04 to 3.81 billion JPY; 15.2; IQR, 7.9–29.0 million USD). Total liabilities also increased, though to a lesser extent, with median liabilities rising from 0.72 to 0.83 billion JPY (6.6 to 6.3 million USD, reflecting exchange rate effects). Medical revenue rose steadily from 11.0 to 12.5 trillion JPY, though the corresponding USD values decreased slightly from 101.1 to 95.1 billion USD due to exchange rate fluctuations. Median revenue increased from 1.43 billion JPY (IQR, 0.82–2.54 billion JPY; 13.1; IQR, 7.5–23.3 million USD) to 1.55 billion JPY (IQR, 0.89 to 2.81 billion JPY; 11.8; IQR, 6.7–21.3 million USD).

**Table 3.** Trends in financial performance indicators among medical corporations, FY2016–FY2022

	FY2016	FY2017	FY2018	FY2019	FY2020	FY2021	FY2022
Medical corporations, *N*	4,631	4,750	4,722	4,656	4,591	4,526	4,469
**Balance sheets**							
Total assets							
Sum, trillion JPY / billion USD	¥13.6 / $125.0	¥14.2 / $126.6	¥14.5 / $131.3	¥14.6 / $133.9	¥14.6 / $136.7	¥15.4 / $140.3	¥15.9 / $121.0
Median (IQR), billion JPY / million USD	¥1.72 (0.89–3.29) / $15.8 (8.2–30.2)	¥1.73 (0.89–3.33) / $15.4 (7.9–29.7)	¥1.77 (0.91–3.45) / $16.0 (8.2–31.2)	¥1.79 (0.92–3.48) / $16.4 (8.4–31.9)	¥1.83 (0.94–3.51) / $17.1 (8.8–32.9)	¥1.94 (1.02–3.70) / $17.7 (9.3–33.7)	¥2.00 (1.04–3.81) / $15.2 (7.9–29.0)
Total liabilities							
Sum, trillion JPY / billion USD	¥7.34 / $67.5	¥7.59 / $67.6	¥7.87 / $71.3	¥7.87 / $72.2	¥7.81 / $73.1	¥8.29 / $75.5	¥8.37 / $63.7
Median (IQR), billion JPY / million USD	¥0.72 (0.28–1.67) / $6.6 (2.6–15.3)	¥0.71 (0.28–1.69) / $6.3 (2.5–15.1)	¥0.73 (0.29–1.74) / $6.6 (2.6–15.8)	¥0.71 (0.28–1.74) / $6.5 (2.6–16.0)	¥0.74 (0.28–1.75) / $6.9 (2.6–16.4)	¥0.81 (0.30–1.86) / $7.4 (2.7–16.9)	¥0.83 (0.32–1.88) / $6.3 (2.4–14.3)
Total net assets							
Sum, trillion JPY / billion USD	¥6.27 / $57.6	¥6.58 / $58.6	¥6.63 / $60.1	¥6.69 / $61.4	¥6.81 / $63.8	¥7.09 / $64.6	¥7.54 / $57.4
Median (IQR), billion JPY / million USD	¥0.72 (0.26–1.61) / $6.6 (2.4–14.8)	¥0.73 (0.26–1.63) / $6.5 (2.3–14.5)	¥0.74 (0.26–1.66) / $6.7 (2.4–15.0)	¥0.75 (0.27–1.66) / $6.9 (2.5–15.2)	¥0.77 (0.27–1.73) / $7.2 (2.5–16.2)	¥0.79 (0.29–1.81) / $7.2 (2.6–16.5)	¥0.85 (0.32–1.95) / $6.5 (2.4–14.8)
Excess liability over assets, *N* (%)	274 (5.9)	315 (6.6)	333 (7.1)	338 (7.3)	351 (7.6)	357 (7.9)	340 (7.6)
**Income statements**							
Medical revenue							
Sum, trillion JPY / billion USD	¥11.0 / $101.1	¥11.4 / $101.6	¥11.7 / $106.0	¥11.9 / $109.2	¥12.0 / $112.4	¥12.1 / $110.2	¥12.5 / $95.1
Median (IQR), billion JPY / million USD	¥1.43 (0.82–2.54) / $13.1 (7.5–23.3)	¥1.43 (0.82–2.54) / $12.7 (7.3–22.6)	¥1.46 (0.83–2.60) / $13.2 (7.5–23.6)	¥1.48 (0.84–2.63) / $13.6 (7.7–24.1)	¥1.50 (0.86–2.71) / $14.0 (8.1–25.4)	¥1.50 (0.87–2.73) / $13.7 (7.9–24.9)	¥1.55 (0.89–2.81) / $11.8 (6.8–21.4)
Medical profit							
Sum, billion JPY / billion USD	¥307.3 / $2.82	¥261.3 / $2.33	¥257.7 / $2.34	¥282.0 / $2.59	¥269.5 / $2.52	¥244.4 / $2.22	¥493.5 / $3.76
Median (IQR), million JPY / million USD	¥24.9 (−13.0 to 94.5) / $0.23 (−0.12 to 0.87)	¥20.1 (−17.0 to 86.7) / $0.18 (−0.15 to 0.77)	¥19.9 (−18.0 to 84.6) / $0.18 (−0.16 to 0.77)	¥18.5 (−19.8 to 86.9) / $0.17 (−0.18 to 0.80)	¥17.4 (−24.1 to 87.8) / $0.16 (−0.23 to 0.82)	¥6.9 (−40.4 to 73.1) / $0.06 (−0.37 to 0.67)	¥19.4 (−29.8 to 102.9) / $0.15 (−0.23 to 0.78)
Medical profit, positive, *N* (%)	3,105 (67.1)	3,027 (63.7)	3,042 (64.4)	2,967 (63.7)	2,829 (61.6)	2,478 (54.8)	2,744 (61.4)
Ordinary revenue							
Sum, trillion JPY / billion USD	¥11.7 / $107.5	¥12.1 / $107.8	¥12.4 / $112.3	¥12.5 / $114.7	¥12.7 / $118.9	¥12.9 / $117.5	¥13.4 / $102.0
Median (IQR), billion JPY / million USD	¥1.51 (0.87–2.69) / $13.9 (8.0–24.7)	¥1.52 (0.87–2.68) / $13.5 (7.8–23.9)	¥1.54 (0.89–2.77) / $13.9 (8.1–25.1)	¥1.56 (0.90–2.80) / $14.3 (8.3–25.7)	¥1.58 (0.92–2.90) / $14.8 (8.6–27.2)	¥1.63 (0.94–2.94) / $14.8 (8.6–26.8)	¥1.67 (0.96–3.09) / $12.7 (7.3–23.5)
Ordinary profit							
Sum, billion JPY / billion USD	¥364.5 / $3.35	¥326.2 / $2.91	¥330.5 / $2.99	¥344.6 / $3.17	¥325.7 / $3.05	¥438.8 / $4.00	¥737.8 / $5.62
Median (IQR), million JPY / million USD	¥34.0 (0.30–106.8) / $0.31 (0.00–0.98)	¥30.6 (−3.62 to 101.9) / $0.27 (−0.03 to 0.91)	¥30.4 (−3.88 to 100.1) / $0.28 (−0.04 to 0.91)	¥28.4 (−6.16 to 103.6) / $0.26 (−0.06 to 0.95)	¥26.5 (−9.48 to 101.1) / $0.25 (−0.09 to 0.95)	¥35.2 (−8.51 to 115.4) / $0.32 (−0.08 to 1.05)	¥47.9 (1.32–157.1) / $0.36 (0.01–1.20)
Ordinary profit, positive, *N* (%)	3,485 (75.3)	3,451 (72.7)	3,447 (73.0)	3,364 (72.3)	3,230 (70.4)	3,224 (71.2)	3,391 (75.9)
Net profit							
Sum, billion JPY / billion USD	¥193.8 / $1.78	¥146.8 / $1.31	¥125.0 / $1.13	¥142.8 / $1.31	¥166.3 / $1.55	¥300.1 / $2.73	¥555.6 / $4.23
Median (IQR), million JPY / million USD	¥21.0 (−6.98 to 73.0) / $0.19 (−0.06 to 0.67)	¥19.4 (−11.2 to 72.6) / $0.17 (−0.10 to 0.65)	¥19.3 (−10.9 to 69.5) / $0.17 (−0.10 to 0.63)	¥18.8 (−12.4 to 73.7) / $0.17 (−0.11 to 0.68)	¥17.7 (−15.1 to 71.9) / $0.17 (−0.14 to 0.67)	¥26.2 (−10.1 to 88.9) / $0.24 (−0.09 to 0.81)	¥35.8 (−0.91 to 120.4) / $0.27 (−0.01 to 0.92)
Net profit, positive, *N* (%)	3,312 (71.5)	3,301 (69.5)	3,286 (69.6)	3,211 (69.0)	3,088 (67.3)	3,182 (70.3)	3,331 (74.5)
**Profitability**							
Medical profit margin, median (IQR), %	1.8 (−1.2 to 5.3)	1.6 (−1.6 to 5.0)	1.5 (−1.7 to 4.8)	1.5 (−1.8 to 4.9)	1.2 (−2.0 to 4.7)	0.6 (−3.4 to 4.3)	1.6 (−2.5 to 5.5)
Ordinary profit margin, median (IQR), %	2.5 (0.0–6.2)	2.3 (−0.4 to 5.8)	2.3 (−0.4 to 5.8)	2.2 (−0.6 to 5.7)	2.0 (−0.7 to 5.3)	2.6 (−0.7 to 6.5)	3.5 (0.1–7.8)
Net profit margin, median (IQR), %	1.6 (−0.6 to 4.4)	1.5 (−1.1 to 4.3)	1.5 (−1.0 to 4.3)	1.5 (−1.1 to 4.3)	1.3 (−1.1 to 4.2)	1.9 (−0.9 to 5.1)	2.7 (−0.1 to 6.2)
Return on total capital from medical business, median (IQR), %	1.7 (−1.0 to 4.5)	1.4 (−1.5 to 4.2)	1.3 (−1.4 to 4.0)	1.3 (−1.5 to 4.0)	1.0 (−1.7 to 3.9)	0.4 (−2.7 to 3.4)	1.2 (−2.1 to 4.3)
Return on total assets (ROA), median (IQR), %	1.5 (−0.6 to 3.7)	1.3 (−1.0 to 3.7)	1.3 (−0.9 to 3.6)	1.3 (−1.0 to 3.6)	1.2 (−1.0 to 3.5)	1.6 (−0.8 to 4.2)	2.2 (−0.1 to 4.9)
Return on equity (ROE), median (IQR), %	3.2 (−0.9 to 7.9)	2.8 (−1.9 to 7.3)	2.6 (−1.7 to 7.2)	2.7 (−1.8 to 7.5)	2.3 (−2.2 to 6.9)	3.1 (−1.3 to 8.5)	4.0 (−0.4 to 10.6)
**Liquidity**							
Current ratio, median (IQR), %	302.4 (154.6–615.7)	300.6 (154.2–625.6)	284.6 (143.8–572.8)	280.0 (137.5–579.0)	289.4 (138.8–619.7)	330.3 (166.3–661.2)	345.6 (174.4–677.7)
**Stability**							
Debt ratio, median (IQR), %	51.2 (25.4–76.3)	49.8 (24.5–76.4)	50.5 (23.9–76.9)	50.0 (22.8–77.0)	49.6 (21.9–77.8)	51.2 (23.1–78.6)	49.4 (22.8–77.1)
Equity ratio, median (IQR), %	48.8 (23.7–74.6)	50.2 (23.6–75.5)	49.5 (23.1–76.1)	50.0 (23.0–77.2)	50.4 (22.2–78.1)	48.9 (21.5–76.9)	50.6 (22.9–77.2)
Debt-to-Equity Ratio, median (IQR), %	83.4 (23.1–239.6)	77.3 (21.5–223.4)	75.2 (21.2–222.0)	73.7 (19.9–222.9)	70.4 (17.6–217.5)	72.9 (17.7–235.0)	71.0 (17.9–217.8)
Fixed asset to long-term liabilities ratio, median (IQR), %	74.1 (57.8–87.7)	74.2 (57.1–87.7)	74.3 (57.8–89.1)	74.9 (57.7–89.6)	74.7 (57.4–89.7)	70.6 (53.4–84.8)	69.3 (52.0–84.2)
**Efficiency**							
Total asset turnover, median (IQR)	0.9 (0.7–1.1)	0.9 (0.6–1.1)	0.9 (0.6–1.1)	0.9 (0.6–1.1)	0.9 (0.6–1.2)	0.8 (0.6–1.1)	0.8 (0.6–1.1)

FY, fiscal year; IQR, interquartile range; JPY, Japanese yen; USD, United States dollar.

All USD values were calculated using the average exchange rate for each fiscal year: 1 USD = 108.8 JPY in FY2016, 112.2 in FY2017, 110.4 in FY2018, 109.0 in FY2019, 106.8 in FY2020, 109.8 in FY2021, and 131.4 in FY2022.

Profit levels showed more variability. Median medical profit declined during the COVID-19 pandemic, reaching a low in FY2021 (6.9 million JPY; IQR, −40.4 to 73.1 million JPY; 0.06; IQR, −0.37 to 0.67 million USD), but recovered to 19.4 million JPY (IQR, −29.8 to 102.9 million JPY; 0.15; IQR, −0.23 to 0.78 million USD) in FY2022. The proportion of corporations reporting positive medical profits decreased from 67.1% in FY2016 to 54.8% in FY2021, then rose to 61.4% in FY2022. In contrast, ordinary and net profits appeared less affected. Ordinary profit remained relatively stable (345 billion JPY in FY2019; 326 billion in FY2020), then increased to 439 billion in FY2021 and 738 billion in FY2022. Net profit increased from 143 billion JPY (1.31 billion USD) in FY2019 to 556 billion JPY (4.23 billion USD) in FY2022.

Profitability indicators showed similar fluctuations. Median medical profit margin decreased from 1.8% (IQR, −1.2 to 5.3%) in FY2016 to 0.6% (IQR, −3.4 to 4.3%) in FY2021, and then rebounded to 1.6% (IQR, −2.5 to 5.5%) in FY2022. By contrast, median ordinary and net profit margins remained relatively stable or improved, reaching 3.5% (IQR, 0.1–7.8%) and 2.7% (IQR, −0.1 to 6.2%) in FY2022, respectively. Median ROA increased from 1.5% (IQR, −0.6 to 3.7%) to 2.2% (IQR, −0.1 to 4.9%), and ROE from 3.2% (IQR, −0.9 to 7.9%) to 4.0% (IQR, −0.4 to 10.6%).

Liquidity, measured by the current ratio, remained stable (302.4% in FY2016; 345.6% in FY2022), as did stability indicators: the median debt ratio and equity ratio in FY2022 were 49.4% (IQR, 22.8–77.1%) and 50.6% (IQR, 22.9–77.2%), respectively. Efficiency, assessed by total asset turnover, remained unchanged at 0.8 to 0.9 (0.8; IQR, 0.6–1.1 in FY2022).

### Subgroup analysis

**Figure 1.**
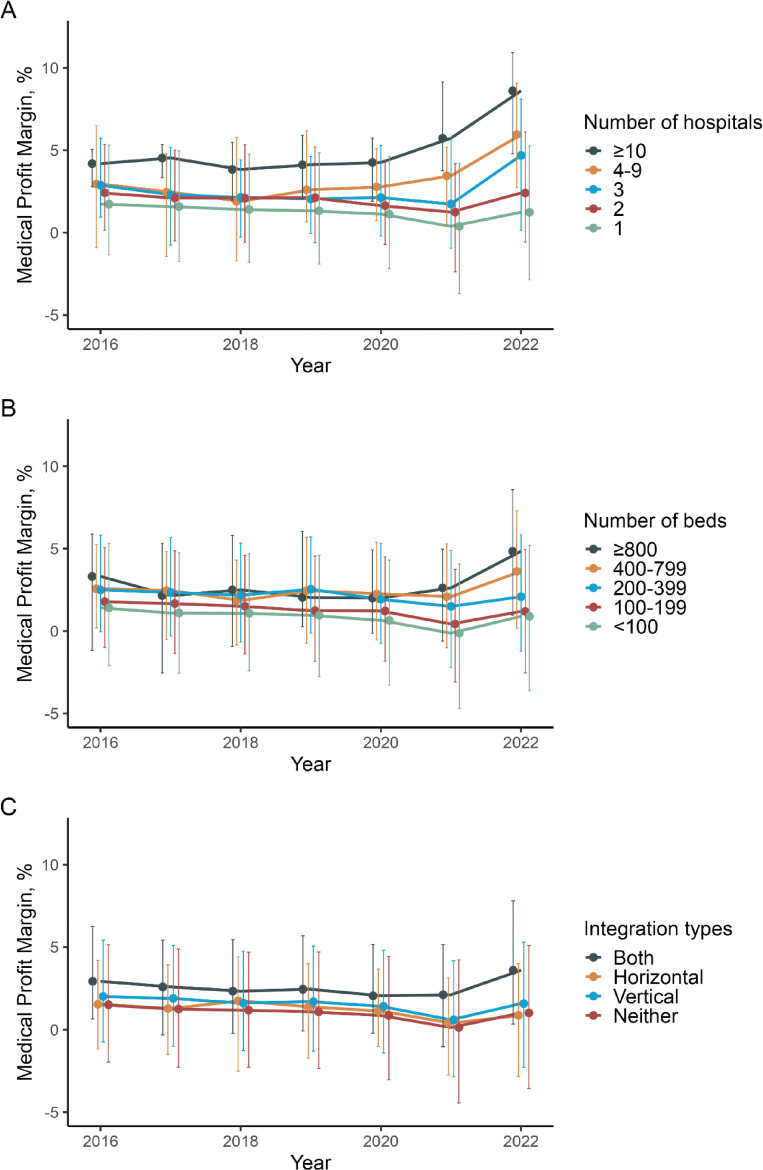
Trends in medical profit margin by organizational characteristics of medical corporations in Japan, FY2016–FY2022. Medical profit margins (%) from FY2016 to FY2022 are presented according to organizational characteristics of medical corporations. (**A**) Median profit margin by number of hospitals operated; (**B**) Median profit margin by total number of beds; (**C**) Median profit margin by integration type. Each point indicates the median value for a given fiscal year, with lines connecting annual medians. Error bars represent interquartile ranges. FY, fiscal year.

Figure 1 displays subgroup trends in medical profit margins from FY2016 to FY2022 by number of hospitals (Figure 1A), number of beds (Figure 1B), and integration types (Figure 1C). Medical corporations with a larger number of hospitals, more beds, or both horizontal and vertical integration consistently exhibited higher profit margins compared to their counterparts. In contrast, corporations with only one hospital, fewer than 100 beds, or no integration tended to have lower profit margins throughout the study period. During FY2020 and FY2021, when the impact of COVID-19 was most pronounced, corporations with fewer hospitals, fewer beds, or no integration experienced a greater decline in profit margins. However, a recovery was observed across all groups in FY2022.

### Association between medical corporation characteristics and profitability

Associations between organizational characteristics and medical profit margin were evaluated using a linear mixed-effects model (Table 4). Model 1 to model 3 sequentially incorporated the number of hospitals, types of beds, and integration types, respectively. In model 1, the number of hospitals showed a positive but non-significant association with medical profit margin (β = 0.41; 95% CI, –0.03 to 0.85; *P* = 0.070). In model 2, the total number of beds was significantly and positively associated with profitability (per 100 beds: β = 0.27; 95% CI, 0.10–0.43; *P* = 0.0014).

**Table 4.** Association between hospital characteristics and medical profit margin: results from a linear mixed-effects model

	Model 1		Model 2		Model 3		Model 4		Model 5		Model 6	
					
	Beta, 95% CI	*P* value	Beta, 95% CI	*P* value	Beta, 95% CI	*P* value	Beta, 95% CI	*P* value	Beta, 95% CI	*P* value	Beta, 95% CI	*P* value
**Variables**												
Number of hospitals	0.41 (−0.03 to 0.85)	0.070									−1.39 (−2.66 to −0.13)	0.031
Number of clinics											0.15 (−0.22 to 0.53)	0.42
Number of long-term care facilities											0.30 (−0.19 to 0.79)	0.23
Number of other facilities											0.27 (−1.01 to 1.56)	0.68
Total beds (per 100 beds)			0.27 (0.10–0.43)	0.0014								
Proportion of general beds					Reference							
Proportion of long-term care beds					0.96 (−0.31 to 2.24)	0.14						
Proportion of psychiatric Beds					0.10 (−1.20 to 1.41)	0.88						
General beds (per 100 beds)							0.08 (−0.15 to 0.31)	0.52			0.46 (−0.05 to 0.96)	0.08
Long-term care beds (per 100 beds)							0.67 (0.22–1.13)	0.0037			0.87 (0.31–1.43)	0.0024
Psychiatric beds (per 100 beds)							0.43 (0.06–0.81)	0.023			0.53 (0.12–0.94)	0.011
Horizontal integration only									1.09 (−1.48 to 3.66)	0.41	1.24 (−1.51 to 3.99)	0.38
Vertical integration only									1.09 (0.17–2.01)	0.021	0.53 (−0.54 to 1.59)	0.33
Both vertical and horizontal integration									2.36 (0.73–4.00)	0.0046	1.74 (−0.30 to 3.77)	0.095
Neither									Reference		Reference	
**Model fit**												
AIC	306,843		306,838		306,843		306,839		306,835		306,839	
BIC	306,927		306,922		306,935		306,940		306,935		306,998	
Marginal R^2^	0.0010		0.0015		0.00098		0.0018		0.0015		0.0025	
Conditional R^2^	0.206		0.206		0.206		0.206		0.206		0.206	

AIC, Akaike Information Criterion; BIC, Bayesian Information Criterion; CI, confidence interval.

Estimates are from a linear mixed-effects model with medical profit margin as the outcome, adjusted for year.

Model 3 is based on bed type proportions, with general beds as the reference category. Models 4 and 6 report coefficients for general, long-term care, and psychiatric beds (per 100 beds).

In model 3, bed type proportions were examined, but neither long-term care (β = 0.96; 95% CI, −0.31 to 2.24; *P* = 0.14) nor psychiatric beds (β = 0.10; 95% CI, −1.20 to 1.41; *P* = 0.88) showed significant associations, with general beds as the reference category. Model 4 revealed that long-term care beds (per 100 beds: β = 0.67; 95% CI, 0.22–1.13; *P* = 0.0037) and psychiatric beds (per 100 beds: β = 0.43; 95% CI, 0.06–0.81; *P* = 0.023) were significantly associated with higher profitability. In model 5, medical corporations with vertical integration showed significantly higher profit margins compared to non-integrated corporations (β = 1.09; 95% CI, 0.17–2.01; *P* = 0.021), and those with both vertical and horizontal integration also had a significant positive association (β = 2.36; 95% CI, 0.73–4.00; *P* = 0.0046). Model 6 included all covariates simultaneously and represents the fully adjusted specification. In this comprehensive model, a greater number of hospitals was significantly associated with a lower medical profit margin (β = −1.39; 95% CI, −2.66 to −0.13; *P* = 0.031), in contrast to the positive coefficient observed in model 1. The number of beds remained positively associated with profitability, particularly long-term care beds (per 100 beds: β = 0.87; 95% CI, 0.31–1.43; *P* = 0.0024) and psychiatric beds (per 100 beds: β = 0.53; 95% CI, 0.12–0.94; *P* = 0.011). Although the coefficients for integration types remained positive, their associations with profit margin were not statistically significant in the fully adjusted model. No serious multicollinearity was observed, with all adjusted GVIF values below 3.0.

## DISCUSSION

### Principal findings

This study presents a comprehensive analysis of financial trends among medical corporations in Japan from FY2016 to FY2022. Although the total number of corporations declined over time, horizontal and vertical integration increased modestly, with a growing proportion managing multiple hospitals or large bed capacities. Despite the COVID-19 pandemic, total assets and medical revenues continued to grow, reaching 15.9 trillion JPY (121.0 billion USD) and 12.5 trillion JPY (95.1 billion USD), respectively, in FY2022, indicating a robust and expanding market. Liquidity and financial stability indicators remained favorable. In contrast, profitability remained modest, and operational efficiency appeared suboptimal. Medical profits declined in FY2020 and FY2021, particularly among smaller corporations owning fewer facilities or beds, whereas larger-scale corporations maintained stable performance. The relatively modest decline in FY2020 compared to FY2021 is difficult to attribute to a single cause with certainty. However, it may reflect the combined effects of fiscal year-end timing, the documented regional variation in the spread and severity of COVID-19 across prefectures in Japan,^13^^,^^14^ and delays in the submission of financial statements by medical corporations due to the pandemic, as acknowledged in official MHLW notices.^15^ These factors suggest that some disruptions may not have been captured in FY2020 but were instead reflected in FY2021, thereby contributing to the observed difference between the two years. However, ordinary and net profit margins were largely unaffected, suggesting that external support, such as COVID-19-related subsidies, may have mitigated the pandemic’s overall financial impact. A linear mixed-effects model indicated that both total bed counts and bed composition were associated with profitability. Specifically, the total number of beds was positively associated with profitability; however, when bed types were entered separately, the associations were stronger for long-term care and psychiatric beds, suggesting that the apparent scale effect was partly driven by the distribution of specific bed types. By contrast, a greater number of hospitals was negatively associated with profitability after adjustment. These findings highlight that both scale and composition play independent roles in shaping financial performance.

### Comparison with other studies

Total medical revenues among Japanese medical corporations continued to grow over time, accompanied by an increase in total assets. According to Organization for Economic Cooperation and Development Data Explorer, Japan’s total health expenditure amounted to 65.6 trillion JPY (466.9 billion USD; exchange rate: 1 USD = 140.5 JPY) in 2023.^16^ Although classifications differ, medical corporations likely account for a substantial share. These findings suggest market expansion and are consistent with international trends showing rising health expenditures across high-income countries.^17^ Our results also indicated that larger-scale corporations maintained higher profitability, while smaller ones were less resilient in terms of profitability to external shocks such as COVID-19. This is aligned with findings from the United States, where smaller hospitals saw greater declines in operating margins, while larger entities demonstrated greater resilience, a trend that has been observed both before and during the COVID-19 pandemic.^18^^–^^21^ In Japan as well, during the pandemic, hospitals increasingly faced difficulties in accepting emergency patients due to shortages of medical staff and protective equipment.^22^ These challenges may have disproportionately affected smaller hospitals, which typically operate with more limited resources. These findings highlight the role of scale in absorbing external financial shocks and maintaining operational continuity.

Subgroup analyses indicated that corporations with more hospitals, more beds, or integration generally exhibited higher medical profit margins. In contrast, the linear mixed-effects model provided an adjusted perspective: after simultaneously accounting for hospital size, bed composition, and integration, a greater number of hospitals was associated with lower profitability, whereas larger bed capacity remained linked to higher margins. Integration status was not significantly associated with profitability in the adjusted model. These findings should be regarded as complementary rather than contradictory. They suggest that, although simple comparisons imply advantages for corporations with multiple hospitals, expansion into several facilities does not necessarily yield efficiency or financial benefits once other structural factors are considered. Given the modest overall fit of the linear mixed-effects model, the results should be interpreted with caution. These findings contrast with studies from the United States, where mergers have often been associated with improved financial performance.^18^^,^^19^^,^^23^ Differences in healthcare system design, regulation, and market structure may explain this discrepancy. In Japan, integration remains limited, and such entities do not necessarily enjoy dominant market positions.^3^ Consequently, operational scale, particularly bed volume, may play a more substantial role in enhancing profitability than integration alone. Prior studies, especially those from the United States, have emphasized hospital size and integration as key determinants of financial resilience. In contrast, our findings highlight the additional importance of bed type composition. In the Japanese context, where long-term care and psychiatric services account for a substantial share of hospital activity, both the type and the number of beds appear to be as relevant as overall scale in influencing profitability.

### Implications

This study found that although revenues among Japanese medical corporations have steadily increased in recent years, profit margins have remained modest. While our findings are broadly consistent with reports from the AHA^4^^,^^5^ and the JHA,^6^ which highlight rising operational costs, such as labor, medical supplies, and utilities, as key constraints on profitability, we were unable to assess cost breakdowns directly due to data limitations. Therefore, caution is warranted in attributing the observed trends to specific cost components. Similarly, a national pulse survey conducted by the United States Department of Health and Human Services Office of Inspector General in February 2021 reported that many hospitals in the United States faced significant financial strain during the COVID-19 pandemic due to increased spending on staffing, protective equipment, and other critical resources.^24^ Collectively, these findings suggest that rising operational costs, compounded by external shocks such as the COVID-19 pandemic, have challenged the sustainability of hospital operations, even amid growing revenues.

Given the current trends, healthcare expenditures in Japan will likely continue to rise, while profitability may remain constrained. From FY2020 to FY2022, disparities in profitability appeared to widen, with corporations owning more facilities or beds demonstrating greater financial resilience. These findings underscore the importance of policy interventions, including targeted subsidies, to support healthcare delivery. However, as previous studies from the United States have noted, indiscriminate financial support may result in overcompensation.^25^ In the present study, although operating profit margins declined during FY2020 and FY2021, ordinary and net profit margins remained stable or improved, suggesting that COVID-19-related subsidies may have been broadly distributed, regardless of need. In FY2022, profits rose sharply despite only modest increases in medical and ordinary revenues. This discrepancy may reflect the impact of targeted medical fee revisions, such as additional charges for acute care hospitalizations, which may have benefited certain corporations depending on their service mix. The continuation of temporary support measures may also have contributed. These factors likely explain the wider variation in profitability observed in FY2022, indicating uneven recovery across institutions. Strategic allocation of financial support is, therefore, essential to ensure that public funds are directed toward institutions most vulnerable to financial distress during public health crises or economic downturns.

Structural reforms, such as hospital mergers, are often considered a means to enhance financial sustainability. In the United States, system affiliation has been associated with reduced financial distress, particularly among smaller or rural hospitals.^18^^,^^19^^,^^23^ Our findings, however, suggest that comparable benefits may not be realized in Japan. Unlike in the United States, where mergers often generate market power and operational efficiencies, Japan’s lower degree of integration and distinct regulatory environment may limit the financial gains from such approaches. These results imply that in Japan, simply increasing the number of hospitals may not improve profitability; instead, merger strategies that also account for the number of beds per hospital and other structural characteristics may be more appropriate. Future research should clarify which types of medical corporations are most financially vulnerable, what forms of support they require, and under what conditions integration is most beneficial. Policymakers need to set clear goals and adopt evidence-based strategies to guide the optimal restructuring of Japan’s healthcare system. In addition to structural reforms and integration strategies, it is also important to recognize the heterogeneity of bed types. Our analyses indicate that long-term care and psychiatric beds, in particular, have stronger associations with profitability than general beds. This suggests that policies aimed at financial sustainability cannot rely solely on expanding scale but must also account for the service mix of hospitals.

### Strengths and limitations of the study

This study has several strengths. First, it used a comprehensive, nationally representative dataset to examine recent structural and financial trends in medical corporations. To our knowledge, this is the first such analysis published in an academic paper, particularly one written in English and accessible to an international audience. While one prior study in Japanese analyzed financial characteristics of medical corporations using data from 1982 to 1991,^26^ no updated or extended analysis has been published since. Our study contributes novel insights based on recent data, facilitating international comparison and informing global health policy discussions. Second, by combining balance sheet and income statement data over multiple years, the study identified longitudinal trends and financial vulnerabilities, particularly during the COVID-19 pandemic. These strengths enabled a detailed assessment of overall financial trends among medical corporations before and during the COVID-19 pandemic, thereby allowing a clearer evaluation of the pandemic’s financial impact.

There are also limitations. First, although the dataset covered approximately 95% of medical corporations, it did not include all institutions. Second, because the data were not collected by government sources, they may contain reporting inaccuracies or missing values. Third, our classification of integration types was based solely on ownership structure. Due to data limitations, we could not distinguish differences in the form or degree of integration, such as shared governance, centralized management, or financial consolidation. These factors may influence financial performance, and future studies should examine them using more detailed organizational data. Fourth, although we did not include medical revenue as an explanatory variable due to its mathematical overlap with the outcome (medical profit margin), this choice may be subject to residual confounding due to income scale. Structural indicators, such as bed capacity and facility count, were used as explanatory variables, but residual confounding cannot be entirely excluded. Fifth, clinical and operational data, including inpatient volumes, staffing levels, and care quality metrics, were not available, limiting our ability to directly interpret financial outcomes in relation to service delivery and patient care. Nevertheless, the increasing proportion of loss-making corporations may indicate vulnerability in maintaining healthcare provision, particularly for smaller organizations and in rural areas, with potential implications for access, continuity, and quality of care. Within the scope of the available data, our findings underscore the importance of considering financial sustainability as an element of health system resilience. Further studies are warranted to integrate financial, clinical, and administrative data to better understand the interrelationships among organizational structures, care processes, outcomes, and financial sustainability.

### Conclusions

Although the number of medical corporations in Japan has gradually declined, the sector’s overall financial scale has expanded, with continued growth in assets and revenues. However, profit margins remained modest, and smaller corporations experienced greater financial strain, especially during the COVID-19 pandemic. These findings underscore the financial vulnerability of smaller institutions during public health emergencies and highlight structural challenges in sustaining profitability across the sector.

## SUPPLEMENTARY MATERIAL

eFigure 1. Classification of medical corporation integration types. Horizontal integration is defined as ownership of multiple hospitals. Vertical integration refers to ownership of a hospital and other facility types such as clinics or long-term care facilities. This figure illustrates the four possible categories: horizontal integration only, vertical integration only, both, and neither.

eFigure 2. Study flow diagram

eTable 1. Distribution of general, long-term care, and psychiatric beds by hospital number, bed size, and integration type
