# Supplementary material for: Financial Performance of Medical Corporations in Japan From 2016 to 2022: A Nationwide Longitudinal Analysis
Source: J Epidemiol. 2026 Jun 5;36(6):188–98. doi: 10.2188/jea.JE20250303 (PMC13158355; doi:10.2188/jea.JE20250303)
Supplement: Supplementary file 1 [file je-36-188-s001.pdf]

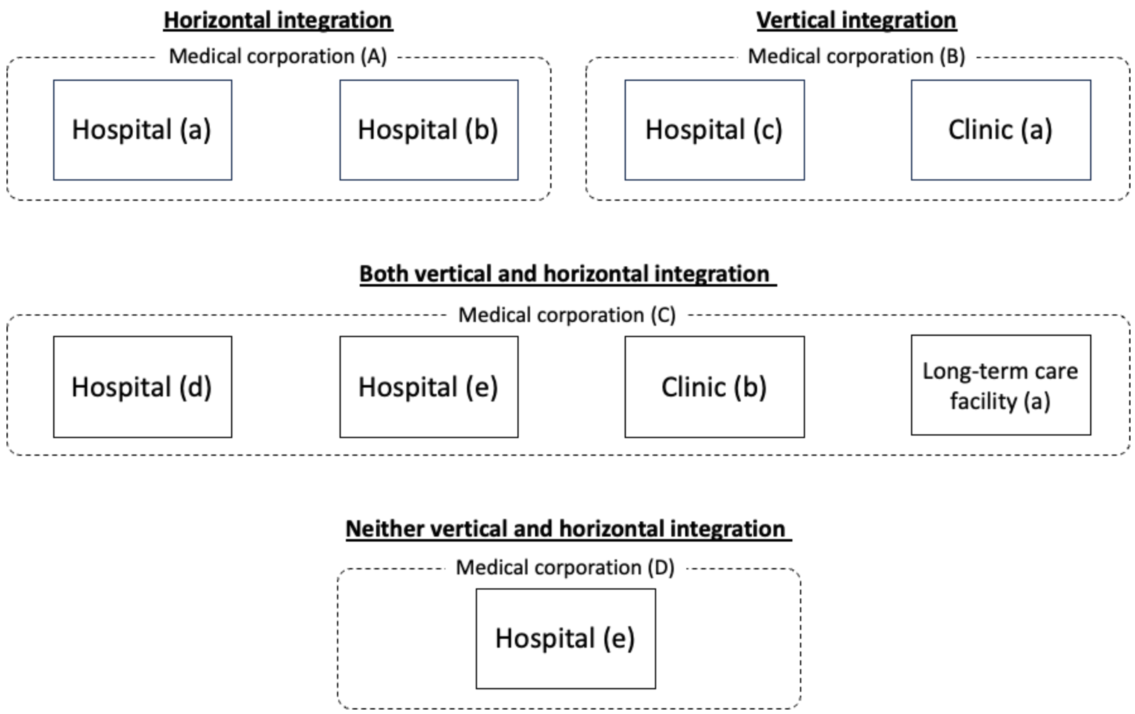

eFigure 1. Classification of medical corporation integration types. Horizontal integration is defined as ownership of multiple hospitals. Vertical integration refers to ownership of a hospital and other facility types such as clinics or long-term care facilities. This figure illustrates the four possible categories: horizontal integration only, vertical integration only, both, and neither.

Base data

	Fiscal year						
	2016	2017	2018	2019	2020	2021	2022
Medical corporation	49366	50787	51409	51960	52211	52178	52021
Facility	62062	64090	65124	66021	66740	67062	67228
Hospital	5479	5661	5649	5622	5559	5480	5415
Clinic	53011	54831	55796	56715	57386	57728	57906
Long-term facility	3107	3179	3188	3133	3140	3081	3045
Other	465	419	491	551	655	773	862

Exclude medical corporations that do not own hospitals

	Fiscal year						
	2016	2017	2018	2019	2020	2021	2022
Medical corporation	44712	46004	46668	47280	47598	47639	47544
							0
Facility	52294	53963	54993	55822	56441	56821	56980
Hospital	0	0	0	0	0	0	0
Clinic	50753	52464	53442	54327	54950	55328	55485
Long-term facility	1166	1169	1158	1128	1129	1111	1094
Other	375	330	393	367	362	382	401

Exclude medical corporations that has outliers in financial indicators

	Year						
	2016	2017	2018	2019	2020	2021	2022
Medical corporation	23	33	19	24	22	13	8
Facility	28	53	31	31	41	23	13
Hospital	23	35	21	26	28	14	9
Clinic	4	9	5	2	7	2	1
Long-term facility	1	8	5	3	6	6	3
Other	0	1	0	0	0	1	0

Include medical corporations that own hospitals

	Fiscal year						
	2016	2017	2018	2019	2020	2021	2022
Medical corporation	4631	4750	4722	4656	4591	4526	4469
Facility	9740	10074	10100	10168	10258	10218	10235
Hospital	5456	5626	5628	5596	5531	5466	5406
Clinic	2254	2358	2349	2386	2429	2398	2420
Long-term facility	1940	2002	2025	2002	2005	1964	1948
Other	90	88	98	184	293	390	461

eFigure 2. Study flow diagram

eTable 1. Distribution of general, long-term care, and psychiatric beds by hospital number, bed size, and integration type

	FY2016				FY2017				FY2018			
	N*	General	Long-term care	Psychiatric	N*	General	Long-term care	Psychiatric	N*	General	Long-term care	Psychiatric
**Category by number of hospitals**												
1	4,118	36.1%	29.9%	34.0%	4,219	36.1%	29.8%	34.1%	4,180	36.2%	29.5%	34.3%
2	385	40.5%	33.1%	26.4%	386	41.0%	32.4%	26.6%	394	41.6%	33.1%	25.3%
3	70	43.1%	32.5%	24.4%	82	39.9%	35.5%	24.6%	80	40.1%	32.8%	27.2%
4–9	54	47.1%	43.0%	9.9%	59	49.7%	40.0%	10.3%	64	50.1%	39.2%	10.6%
≥10	4	78.6%	15.6%	5.8%	4	78.4%	15.9%	5.7%	4	78.1%	16.5%	5.5%
**Category by number of beds**												
<100	1,911	58.3%	39.1%	2.6%	1,950	58.6%	38.9%	2.5%	1,930	58.5%	39.2%	2.4%
100–199	1,483	40.4%	36.7%	22.9%	1,538	40.8%	36.2%	23.1%	1,525	40.9%	35.8%	23.3%
200-–399	903	29.9%	25.3%	44.7%	917	29.4%	25.6%	45.0%	917	30.0%	25.6%	44.4%
400–799	278	30.7%	26.2%	43.1%	283	31.7%	25.6%	42.7%	285	32.2%	24.7%	43.1%
≥800	56	49.5%	31.5%	19.0%	62	49.1%	31.1%	19.8%	65	49.4%	31.1%	19.6%
**Integration types**												
Horizontal	129	29.9%	35.6%	34.5%	135	31.4%	35.2%	33.4%	142	32.8%	36.2%	31.1%
Vertical	1,835	36.6%	30.3%	33.0%	1,876	36.5%	30.2%	33.3%	1,871	36.8%	29.5%	33.7%
Both	384	49.2%	33.4%	17.3%	396	49.3%	33.0%	17.7%	400	49.7%	32.5%	17.7%
Neither	2,283	35.4%	29.5%	35.1%	2,343	35.7%	29.3%	35.0%	2,309	35.5%	29.5%	35.0%

	FY2019				FY2020				FY2021			
	N*	General	Long-term care	Psychiatric	N*	General	Long-term care	Psychiatric	N*	General	Long-term care	Psychiatric
**Category by number of hospitals**												
1	4,111	36.2%	28.8%	35.0%	4,055	36.9%	28.1%	35.1%	3,991	37.4%	26.9%	35.8%
2	391	41.9%	31.7%	26.4%	384	43.6%	29.6%	26.8%	380	44.3%	29.4%	26.4%
3	85	39.9%	34.9%	25.2%	80	39.0%	34.0%	26.9%	84	39.6%	31.5%	28.9%
4–9	63	50.7%	38.4%	10.9%	66	50.1%	38.0%	11.9%	65	51.2%	37.1%	11.7%
≥10	6	76.0%	19.4%	4.6%	6	75.5%	19.1%	5.4%	6	75.8%	18.3%	5.9%
**Category by number of beds**												
<100	1,908	58.7%	39.1%	2.2%	1,898	59.2%	38.5%	2.3%	1,866	59.6%	38.0%	2.4%

100–199	1,513	40.5%	35.7%	23.9%	1,495	41.0%	34.5%	24.4%	1,475	42.0%	33.2%	24.8%
200–399	895	30.2%	24.8%	45.0%	858	30.4%	24.2%	45.4%	853	30.5%	23.1%	46.4%
400–799	271	30.9%	23.0%	46.1%	274	32.5%	21.8%	45.7%	266	33.0%	21.3%	45.7%
≥800	69	52.6%	29.6%	17.8%	66	53.4%	28.5%	18.0%	66	54.8%	26.5%	18.8%
**Integration types**												
Horizontal	130	34.6%	33.2%	32.2%	124	35.2%	30.3%	34.5%	124	35.7%	29.6%	34.7%
Vertical	1,870	37.4%	28.8%	33.7%	1,909	37.9%	28.8%	33.2%	1,902	38.5%	28.1%	33.5%
Both	415	49.8%	32.4%	17.8%	412	50.5%	31.3%	18.2%	411	51.2%	30.4%	18.4%
Neither	2,241	34.9%	28.7%	36.4%	2,146	35.7%	27.2%	37.1%	2,089	36.1%	25.5%	38.4%

	FY2022			
	**N[*]**	**General**	**Long-term care**	**Psychiatric**
**Category by number of hospitals**				
1	3,941	37.4%	26.3%	36.3%
2	373	44.4%	28.9%	26.6%
3	82	41.8%	30.8%	27.3%
4–9	67	52.8%	35.7%	11.5%
≥10	6	73.9%	21.5%	4.6%
**Category by number of beds**				
<100	1,864	59.2%	38.5%	2.3%
100–199	1,445	42.3%	32.4%	25.3%
200–399	831	30.5%	21.7%	47.8%
400–799	264	33.4%	21.8%	44.8%
≥800	65	56.1%	26.3%	17.6%
**Integration types**				
Horizontal	125	35.3%	30.1%	34.6%
Vertical	1,905	38.8%	27.5%	33.7%
Both	403	52.4%	30.0%	17.6%
Neither	2,036	35.8%	24.8%	39.4%

FY, fiscal year.
*N indicates the number of medical corporations.
